# Bacteria Patterns on Tonsillar Surface and Tonsillar Core Tissue among Patients Scheduled for Tonsillectomy at Bugando Medical Centre, Mwanza, Tanzania

**DOI:** 10.3390/pathogens10121560

**Published:** 2021-11-30

**Authors:** Gustave Buname, Gapto Aristides Kiwale, Martha F. Mushi, Vitus Silago, Peter Rambau, Stephen E. Mshana

**Affiliations:** 1 Department of ENT, Bugando Medical Centre, Weill Bugando School of Medicine, Catholic University of Health and Allied Sciences, Mwanza P.O. Box 1464, Tanzania; gbuname7@gmail.com; 2 Department of Microbiology and Immunology, Weill Bugando School of Medicine, Catholic University of Health and Allied Sciences, Mwanza P.O. Box 1464, Tanzania; aristidesgaptoninc@gmail.com (G.A.K.); vsilago.silago2@gmail.com (V.S.); stephen72mshana@gmail.com (S.E.M.); 3 Department of Pathology, Weill Bugando School of Medicine, Catholic University of Health and Allied Sciences, Mwanza P.O. Box 1464, Tanzania; ra1972tz@yahoo.com

**Keywords:** tonsillar, Tanzania, core, surface, tonsillectomy, bacteria

## Abstract

Background: Tonsillitis is an inflammation of the tonsils due to either viruses or bacteria. Here, we report the bacteria patterns on the tonsillar surface and tonsillar core tissue among patients scheduled for tonsillectomy at Bugando Medical Centre (BMC), Mwanza Tanzania. Methods: The study included 120 patients planned for tonsillectomy between April and July 2019. Swab samples from tonsillar surface pre-tonsillectomy and core post-tonsillectomy were collected. Culture was performed following the microbiology laboratory standard operating procedures. Data analysis was completed using STATA version 13, as per the study objectives. Results: The slight majority of participants were males (73; 60.83%) with median age of 6 years (interquartile range 4–11). The proportion of positive culture growth was higher on the surface than in core swab samples: 65 (54.2%) vs. 42 (35.0%), *p* = 0.003. The commonest bacterial pathogen detected from the surface and core were *S. aureus* in 29 (40.3%) and 22 (51.2%) participants, followed by *S. pyogenes* in 17 (23.6%) and 11 (25.6%), respectively. Methicillin-resistant *Staphylococcus aureus* (MRSA) was observed in 20/51 (39%) of isolates. *Streptococcus pyogenes* resistance to macrolides ranged from 8.3% for core isolates to 35.3% for surface isolates. Features suggestive of tonsillitis on histology were reported in 83 (73.5%) samples. Conclusion: More than two-thirds of patients undergoing tonsillectomy had a positive culture for possible bacterial pathogens. *Staphylococcus aureus* and *Streptococcus pyogenes* were the predominant bacteria detected with more than one third of *Staphylococcus aureus* being MRSA. More studies to investigate the treatment outcome of these patients are highly recommended.

## 1. Introduction

Tonsillectomy is one of the most frequently performed otolaryngological operation worldwide [1]. Tonsillectomy can be performed at any age but is mainly performed among children [2,3]. Recurrent acute tonsillitis, with at least five or more attack episodes in a year, obstructive sleep apnea and peritonsillar abscess, is indicated in patients with adenotonsillar hypertrophy [4]. Recurrent acute tonsillitis is mainly associated with bacterial and/or viral infection of the tonsillar crypts or parenchyma, and is characterized by sore throat, fever, odynophagia, and leukocytosis, accompanied by congested tonsils with or without enlargement and tender jugulodigastric lymph nodes. Tonsil infection may occur primarily or secondarily as a result of upper respiratory tract infections commonly preceded by viral infections [5]. Several pathogens are implicated in tonsil infections including: Group A beta hemolytic Streptococcus, alpha hemolytic Streptococcus, *Hemophilus influenzae*, *Staphylococcus aureus*, *Enterococcus* spp., *Klebsiella pneumoniae*, *Brahmnella catarrhalis*, *Corynebacterium* spp. and anaerobes-like Peptostreptococci, Fusobacterium and Veillonella [6,7]. Nevertheless, previous studies conducted worldwide reported the possibility of the tonsillar surface being colonized by the microbial flora which are not implicated in the tonsil infections [6,8]. The bacteria on the core of the tonsils are highly associated with tonsillitis and play a vital role in the recurrent nature of the infections [9]. Furthermore, the recurrent and chronic nature of tonsillitis is highly associated with the ability of the implicated pathogen to resist antibiotic therapy. Antibiotic resistance is reported to be highly linked with the low concentration of antibiotics in the tonsilar core tissue caused by scarring as a result of recurrent infections which impair the antibiotic diffusion [5,10,11]. Penicillin, resistant among pharyngeal flora, also accounts for the recurrent and chronic nature of tonsillitis [12]. In Tanzania, penicillin resistance is reported to range from 53% to 67.8% among *S. pneumoniae* [13,14] one of the pathogens that causes tonsillitis. The steady increase in resistance is reported to be associated with irrational use of the antibiotic in the community [15].

The significant complications of tonsillitis in resource-limited settings is attributed to poor or lack of evidence-based and empiric antibiotic treatment guidelines, due to the lack of data from local studies [16]. Recurrent tonsillitis poses a great health risk to patients including the post streptococcus (Rheumatic heart disease (RHD)) diseases. The RHD is believed to be more prevalent in resource-limited settings such as in Tanzania [17]. The effective treatment of recurrent tonsillitis highly depends on the ability to isolate the associated pathogen and its susceptibility pattern. The primary aim of this study was to determine patterns of Gram-positive bacteria on the tonsillar surface pre tonsillectomy and tonsillar core tissue post tonsillectomy, among patients scheduled for tonsillectomy at Bugando Medical Centre (BMC), Mwanza, Tanzania, in order to obtain data to guide the empirical treatment of tonsillitis.

## 2. Results

A total of 120 patients scheduled for tonsillectomy were selected. The median age of the studied patients was 6 years (interquartile range (IQR) 4–11). The majority of the studied patients were male 73 (60.8%) and the majority of the patients’ parents were from urban areas (98; 81.7%), Table 1.

A total of 114 (95%) of the studied patients experienced breathing difficulties and 60 (50%) had a fever. About one-quarter 31 (25.8%) of the studied patients had a history of hospital admission and three-quarters (90; 75%) had a history of antibiotic use due to tonsillitis. The majority of participants reported the antibiotic used was decided by the health care workers (64; 71.1%), Table 2.

**Figure 1:**
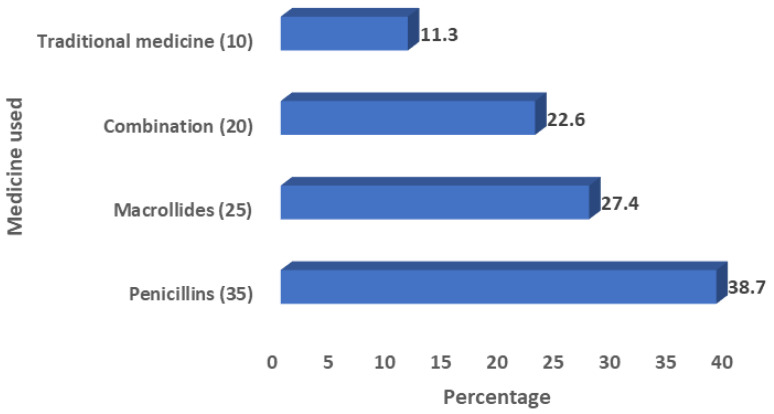
Type of medication used before tonsillectomy.

Out of 90 patients with a history of antibiotic use, 26 (28.9%) had a self-prescription. A total of 29 (24.2%) patients were aware of antimicrobial resistance. More than one-third of patients reportedly used penicillin (35; 38.7%), while macrolides were reported by more than one-quarter 25 (27.4%). A total of 10 (11.3%) patients reportedly used traditional medicine for tonsillitis before tonsillectomy (Figure 1).

Recurrent tonsillitis was reported in 99 (82.5%) patients. The indication for tonsillectomy included; combination of obstructive sleep apnea, throat pain and swallowing difficulty (82; 68%), a single reason of obstructive sleep apnea (28; 23.3%), throat pain (8; 6.7%) and swallowing difficulty (2; 1.7%).

### 2.1. Culture Results Per Recurrence Status

A total of 86 (71.7%) patients had a positive culture growth of possible pathogenic bacteria on either the tonsillar surface or tonsillar core. Of 86 patients with a positive bacteria growth, 67 (77.9%) reportedly had recurrent tonsillitis. A further sub-analysis revealed that, the pathogens from the patients (n = 99) with recurrent tonsillitis were the same as those in patients (n = 21) with no recurrence. *S. aureus* and *S. pyogenes* were predominant pathogens. Significantly more growth of possible pathogens was observed in patients with no history of recurrent tonsillitis than in those with recurrent tonsillitis (19/21 (90.5%) vs. 67/99 (67.7%), *p* = 0.035).

### 2.2. Culture and Susceptibility Patterns of Bacteria from the Tonsillar Core and Surface

The proportion of positive culture growth was significantly higher on surface swab samples than in core swab samples: 65 (54.2%) vs. 42 (35.0%); *p* = 0.003. The prevalence of group A Streptococcus (*Streptococcus pyogenes*) was found to be 14.2% (17/120). In 65 surface swabs, there were seven patients with dual bacteria growth, creating a total of 72 isolates. *S. aureus* (29; 40.3%) followed by *S. pyogenes* (17; 23.6%) were the most predominant, possible, pathogenic bacteria isolated on the tonsil surface, Table 3. In 42 core swabs, there was one patient with dual bacteria growth, creating a total of 43 isolates. *S. aureus* (22; 51.2%) followed by *S. pyogenes* (12; 27.9%) were the most predominant, possible, pathogenic bacteria isolated in the tonsil core, Table 3. *S. pyogenes* and *S. pneumoniae* were 100% sensitive to penicillin and vancomycin in all surface and core isolates. Methicillin-resistant *Staphylococcus aureus* (MRSA) was observed in 20/51 (39%) of isolates.

### 2.3. Histology Results

**Figure 2:**
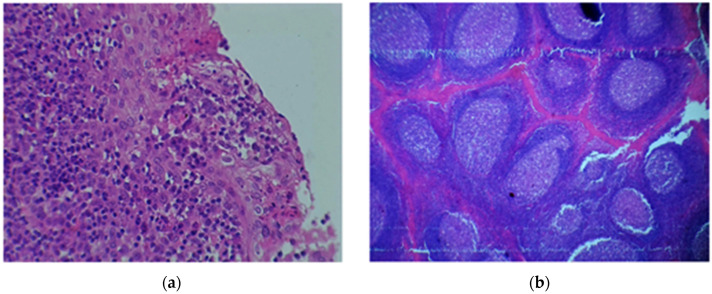
(**a**) Hematoxylin and eosin tissue stain showing tonsillar squamous epithelium infiltrated by lymphocytes × 40. (**b**) Hematoxylin and eosin tissue stain showing variably sized follicles proliferation with prominent germinal centers, and fibrous tissue between the follicles × 4.

**Figure 3:**
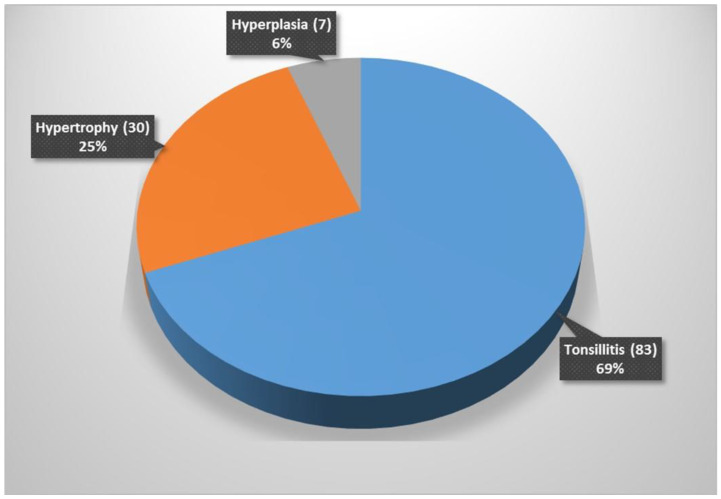
Histology results if tonsillar tissue.

Histological examinations of the 120 tonsillar tissues indicated that 83 (69%) had features suggestive of tonsillitis (Figure 2a,b). Seven (6%) tissues had features of reactive follicular tissue hyperplasia (Figure 3). From 83 tissues samples with suggestive features of tonsillitis, 68 (79.1%) had a positive growth of possible, pathogenic bacteria. A significantly high proportion of samples with features suggestive of tonsillitis in histology had a higher pathogenic bacteria growth than those without features suggestive of tonsillitis in histology: 68 (79.1%) vs. 18 (20.9%); *p* < 0.001. Furthermore, a high proportion of tissue with suggestive features of tonsillitis had a higher positive growth of possible pathogenic bacteria on the surface than in the core: 64 (90.1%) vs. 31 (43.6%); *p* < 0.001. A total of 27 (38.5%) tissues had a positive growth of possible pathogenic bacteria on both surface and core swabs.

Tonsillar tissue, with features suggestive of tonsillitis in histological examinations, had a 4.8 probability of having positive culture results for possible pathogenic bacteria, compared to tonsillar tissue without features suggestive of tonsillitis (OR 4.8, 95% CI 2.0–11.2, *p* < 0.001).

## 3. Discussion

The local epidemiological data for bacteria in the core and surface of the tonsils of patients clinically diagnosed with tonsillitis are important for establishing the proper empirical management protocol of these patients. In response to the call of the World Health Assembly for global action to combat RHD in 2018, this study ‘s contribution was to provide data on the patterns of bacteria found on the surface and core of tonsils among patients scheduled for tonsillectomy. Nearly half of the patients in the current study were found to have possible pathogenic bacteria on the tonsillar surface with the predominance of *Staphylococcus aureus* and *Streptococcus pyogenes*. Furthermore, we observed that more than one-third of *Staphylococcus aureus* isolates were resistant to methicillin, signifying the importance of local susceptibility data in guiding the antibiotic treatment for patients with tonsillitis.

The findings from this study are significantly different from the study in Iraq involving 73 children in the age range of 3–10 years, which reported a prevalence of 58.9% [3]. Furthermore, this prevalence was significantly higher than 15.9%, as the previously reported figure from a study conducted in Uganda [17]. The broad range of possible pathogens reported in the current study could explain this difference, since the study conducted in Uganda was centered on only group A *Streptococcus* spp. The prevalence of group A *Streptococcus* spp. observed in the current study is similar to what was reported in Uganda signifying the importance of this pathogen in the pathogenesis of tonsillitis [17].

The current study found the prevalence of possible pathogenic bacteria in tonsillar core to be 35.0%. This prevalence is almost similar to 40.7% which was previously reported in India in the age group between 11 and 20 years [5]. In addition, the prevalence observed in this study was significantly lower than the 90.4% reported from a study conducted in Iraq [3]. These differences could be explained by the nature of the sample and geographical differences. The study in Iraq used fine-needle aspiration, which has higher ability to concentrate the pathogen and increase the yield of the pathogen than the core swab used in the current study. The fine needle aspiration culture is reported to have a 100% sensitivity, which is higher than the 82.9–92% sensitivity of the core swab culture [10,18].

As previously reported elsewhere [3,5,18], the current study found a mixed growth of possible pathogenic bacteria in seven tonsil surface swabs and one core swabs. The type of bacteria species on the surface and in the core of the tonsils was similar in 22.1% of studied patients. This was also reported from previous studies [10,18].

*Staphylococcus aureus* and *Streptococcus pyogenes* were the predominant bacteria isolated on both the tonsil surface and in the tonsil core. This was previously reported from other studies [5,10]. Methicillin-resistant *Staphylococcus aureus* (MRSA) was observed in 39% of isolates. This was within the range (28.6–50%) of MRSA reported by different studies conducted in the same setting among patients with different infections [19,20,21]. A similar resistance trend was reported in India [5].

## 4. Limitations

The inability to perform anaerobic cultures of bacteria and tissue cultures for viruses might lead to an underestimation of the reported prevalence. The use of patients scheduled for tonsillectomy limits the reproducibility of the study results to the general population of patients with tonsillitis. The study cannot give a detailed insight into the type and composition of traditional medicine used by patients as the data were not collected.

## 5. Conclusions and Recommendations

More than two-thirds of patients undergoing tonsillectomy at BMC had a positive culture growth of possible pathogenic bacteria. *Streptococcus pyogenes* and *Staphylococcus aureus* were the predominant bacteria detected with more than one-third of *Staphylococcus aureus* being MRSA. More studies to investigate the treatment outcome of these patients are highly recommended.

## 6. Methodology

This prospective, cross-sectional, hospital-based study was conducted among patients scheduled for tonsillectomy between April and July 2019. The study was conducted in the otolaryngology department of BMC, a tertiary teaching hospital of the Catholic University of Health and Allied Health Sciences, which has a bed capacity of 1000. The otolaryngology department of BMC performs about 70–80 tonsillectomies each month. The minimum sample size was obtained using a Kish Leslie formula (1965) with a 75% prevalence of tonsillitis from a study conducted in India, which analyzed the patterns of bacteria from patients with tonsillitis [16].

Patients were serially recruited to the study until the sample size was reached. The study excluded all patients with an indication of a tumor as judged by the attending clinicians. Data (age, gender, employment status, and history of breathing difficulty, fever, hospital admission, dysphagia, history of family members with tonsillitis, antibiotic usage in the past two months and recurrent tonsillitis, etc.) were obtained using a pre-coded structured questionnaire before the patient was anesthetized.

Tonsils were defined as the lymphoepithelial structures that provide a protective immunological ring at the openings of both the digestive and respiratory tracts [22]. In the current study, recurrent tonsillitis was defined by having more than five episodes a year [23]. Tonsil core was defined as the inner part of the tonsil, including the tonsil crypt, which was reached by dissecting the tonsil tissue.

### 6.1. Sample Collection and Processing

The surface of the tonsil was rubbed using sterile swab by the experienced surgeon while patient is under anesthesia. The tonsillectomy was conducted followed by the dissection of the tonsil to expose the core area and a second swab was taken immediately. All swabs were placed in the Stuart transport media and transported to microbiology laboratory for processing within two hours of collection. All microbiological analyses were conducted at the microbiology research laboratory of CUHAS following standard operating procedures.

Swabs were inoculated on 5% sheep blood agar and chocolate agar. The plates were incubated in the candle jar for 18–24 h at 37 °C. The identification of bacteria species was based on their growth morphology, hemolytic patterns in blood agar and biochemical/physiological tests such as catalase, coagulase, bile solubility test, and their sensitivity patterns to bacitracin, optochin and trimethoprim/sulphamethoxazole. Antimicrobial susceptibility testing was conducted using Kirby-bauer disc diffusion technique for 15 µg erythromycin(E), 10 µg linezolid(LZD), 10µg gentamicin(CN), 30 µg amikacin(AK), 1.25/23.75 µg trimethoprim-sulfamethoxazole(SXT), 5 µg ciprofloxacin(CIP) and 2 µg clindamycin(DA) [24]. Cefoxitin(FOX) discs (30µg) were used for detection of MRSA as per Clinical Laboratory Standard Institute (CLSI) recommendations [25].

### 6.2. Histological Analysis

The excised tonsil was immediately immersed in the buffered formalin for complete fixation then transferred to histology laboratory for processing by an experienced technologist, and then read by a pathologist. In the histology laboratory, the tissue was processed, sectioned and stained by hematoxylin and eosin as previously documented [26].

### 6.3. Data Analysis

Data were entered on Excel spreadsheet for consistent checking and cleaning, and then transferred to STATA version 13 for analysis. Categorical data were summarized using proportions while continuous data were summarized using median and interquartile ranges. Frequency run was conducted to determine the proportions while two proportion test was conducted to determine the level of differences in microbiological and histological results. P value of less than 0.05 at 95% confidence interval was considered statistically significant.

### 6.4. Ethical Considerations

The protocol of this study was ethically approved by the CUHAS/BMC research ethics and review committee (CREC) with certificate number 912/2019. For participants aged under 18 years, informed consent from a parent and/or legal guardian was obtained before selection. All the procedures were performed following the ethical guidelines.

## Figures and Tables

**Table 1:** Social demographic and clinical data of 120 studied patients.

Variable	Median/Frequency	* IQR/Percentage (%)
**Age**	6	4–11
**Sex**		
Male	73	60.8
Female	47	39.2
**Parents residence**		
Rural	22	18.3
Urban	98	81.7
**Parents Education level**		
Uneducated	3	2.5
Primary	19	15.8
Secondary	52	43.3
University	46	38.3

* IQR: Interquartile range.

**Table 2:** Clinical characteristics and antibiotic use among study participants.

Variable	Frequency	Percentage
**Breathing difficulty**		
Yes	114	95.0
No	6	5.0
**Fever**		
Yes	60	50.0
No	60	50.0
**Tonsillitis infection history in family**		
Yes	14	11.7
No	106	88.3
**Previous operation?**		
Yes	11	9.2
No	109	90.8
**History of hospital admission**		
Yes	31	25.8
No	89	74.2
**Recurrent tonsillitis**		
Yes	99	82.5
No	21	17.5
**Dysphagia**		
Yes	93	77.5
No	27	22.5
**Antibiotic usage in past two months**		
Yes	90	75.0
No	30	25.0
**Antibiotic usage decision**		
Health care worker	64	71.1
Self	26	28.9
**Health care seeking**		
On symptom insert	64	53.3
Several days post infection	32	26.7
After drug failure	24	20.0
Patients AMR * awareness		
Yes	29	24.2
No	91	75.8

* AMR is antimicrobial resistance.

**Table 3:** Sensitivity patterns of Gram-positive bacteria isolated from the surface and core tonsillar swab.

Bacteria Isolated (n)	E n(%)	DA n(%)	LZD n(%)	SXT n(%)	CIP n(%)	CN n(%)	AK n(%)	FOX n(%)
**Surface**								
*S. aureus* (29)	14 (48.3)	18 (62.1)	4 (13.8)	5 (17.2)	15 (51.7)	18 (62.1)	23 (79.3)	18 (62.1)
*S. pyogenes* (17)	11 (64.7)	12 (70.6)	10 (58.8)	0 (0.0)	11 (64.7)	14 (82.4)	14 (82.4)	
*S. viridans* (15)	2 (13.3)	13 (86.7)	14 (93.3)	2 (13.3)	10 (66.7)	11 (73.3)	12 (80)	
*S. pneumoniae* (4)	3 (75)	3 (75)	3 (75)	3 (75)	2 (50)	2 (50)	2 (50)	
*Enterococcous* spp. (3)	2 (66.7)	2 (66.7)	2 (66.7)	1 (33.3)	3 (100)	3 (100)	2 (66.7)	
**Core**								
*S. aureus* (22)	9 (40.9)	12 (54.5)	12 (54.5)	8 (36.4)	13 (59.1)	14 (63.6)	15 (68.2)	13 (59.1)
*S. pyogenes* (12)	11 (91.7)	6 (50.0)	9 (75.0)	0 (0.0)	8 (66.7)	9 (75.0)	9 (75.0)	
*S. viridans* (6)	3 (50.0)	5 (83.3)	4 (66.7)	1 (16.7)	2 (33.3)	3 (50.0)	3 (50.0)	

E—erythromycin, DA—clindamycin, LZD—linezolod, SXT—trimethoprim-sulfamethoxazole, CIP—ciprofloxacin, AK—amikacin, FOX—cefoxitin, CN-Gentamicin.

## Data Availability

The datasets used and/or analyzed during the current study are available from the corresponding author upon reasonable request.
